# Drug-delivering devices in the urinary tract: A systematic review

**DOI:** 10.1080/2090598X.2021.1885948

**Published:** 2021-03-03

**Authors:** Panagiotis Kallidonis, Constantinos Adamou, Sara Villarrova Castillo, Despoina Liourdi, Evangelos Liatsikos, Dirk Lange

**Affiliations:** a Department of Urology, University Hospital of Patras, Patras, Greece; b Endourological Society, Ureteral Stent Research Group; c Valencia, Spain; d General Hospital of Aghios Andreas,Patras, Greece; e Department of Urologic Sciences, University of British Columbia, Vancouver, BC, Canada

**Keywords:** Drug-eluting stents, drug-coated balloons, stenosis, encrustation

## Abstract

**Objective:**

: To systematically review the use of drug-eluting stents (DES) and drug-coated balloons (DCB) in urology.

**Materials and Methods:**

The systematic review was conducted according to the Preferred Reporting Items for Systematic Reviews and Meta-Analyses (PRISMA) statement. PubMed, Scopus, Web of science and Cochrane Library online databases were searched in February 2019. Experimental and clinical studies, which included the placement of a DES or dilatation with DCB for investigating their potential use in the urinary tract for the management of ureteric or urethral pathologies, were included. The primary endpoint was to evaluate the current use of DES and DCB in urology.

**Results:**

A total of 29 articles were included in the systematic review. A total of 10 studies tested DES or DCB containing anti-proliferative agents (paclitaxel, zotarolimus, sirolimus, halofugione). Antibiotic agent-containing DES were tested in nine studies (triclosan, quinolones, teicoplanin, nitrofurantoin, silver sulfadiazine). A total of eight studies investigated the release of anti-inflammatory agents by DES (ketorolac, indomethacin, EW-7197). Another group studied heparin-eluting stents.

**Conclusion:**

Despite the inconclusive outcomes of the three randomised controlled trials, drug-coated/eluting devices constitute a promising field in urology for the prevention of complications associated with conventional stents including pain and encrustation. Pre-clinical *in vitro* and *in vivo* studies have shown their ability to mitigate inflammation, inhibit re-stenosis and improve pain as indicated by declined use of anti-inflammatory drugs.

**Abbreviations**: DES: drug-eluting stents; DCB: drug-coated balloons; DCS: drug-coated stents; HF: halofungione; MCP-1: monocyte chemoattractant protein 1; PRISMA, Preferred Reporting Items for Systematic Reviews and Meta-Analyses; PTCA: percutaneous transluminal coronary angioplasty; RANTES: regulated on activation, normal T-cell expressed and secreted; RCT: randomised controlled trial; USSQ, Ureteric Stent Symptoms Questionaire.

## Introduction

The ureteric stent is a useful tool to avoid kidney damage or sepsis in cases of obstructive uropathy due to stenoses, stones, and extrinsic compression. The ideal ureteric stent should demonstrate optimal flow characteristics and should be well tolerated by the patient, but conventional stents have significant comorbidities, including stent-associated infection, encrustation, migration, hyperplastic urothelial reaction, and patient’s discomfort [1].

Another frequent condition affecting the lower urinary tract is urethral stricture formation related to high re-stenosis rates. A minimally invasive treatment for this is mechanical dilatation with a balloon or placing a urethral stent [2], which are fraught with the same problems as ureteric stents, with rates of re-stenosis despite stent-placement being relatively high [3].

Over recent years, the development of strategies to address ureteric stent-associated complications has been driven by progress made in cardiology. This is mostly due to the fact that vascular stenting, much like the ureter and urethra, involves maintaining patency of a tube whose main function is to conduct a liquid, and innovation in the cardiovascular space to prevent stenosis has progressed faster than in urology. In cardiology, percutaneous transluminal coronary angioplasty (PTCA) has become the main method of coronary re-vascularisation; however, re-stenosis remains a significant complication. Devices that release immunosuppressive agents to minimise benign tissue proliferation, which characterises intimal hyperplasia, proved to be successful over the years. These drug-eluting stents (DES) are devices that release a single or multiple bioactive agents in a controlled manner, depositing the agent on adjacent tissues, while drug-coated stents (DCS) are covered with a single or multiple pharmaceutical agents that provides additional properties to the stent [4]. These devices are widely used in cardiology and vascular surgery, and have significantly reduced the re-stenosis rates after PTCA [5,6]. A more recent approach to further reduce re-stenosis rates after PTCA is the use of drug-coated balloons (DCB) that release the immunosuppresive agent directly at the site of the dilatated vascular stricture and reduce the re-stenosis rates without the need for an indwelling foreign material (i.e. stent) postoperatively. These balloons have reduced the need for DES and can also treat vascular sites that were otherwise unsuitable for stent insertion [7].

The high success of the DES in cardiology and interventional radiology led to the adoption of the drug-eluting concept by endourological research in an attempt to prevent significant complications associated with indwelling ureteric stents [8]. The concept of DCB for use in endourology has been proposed.

The present systematic review aimed to evaluate the literature and provide information about the use of DRE and DCB in urology.

## Methods

### Search strategy – Eligibility criteria

**Figure 1.**
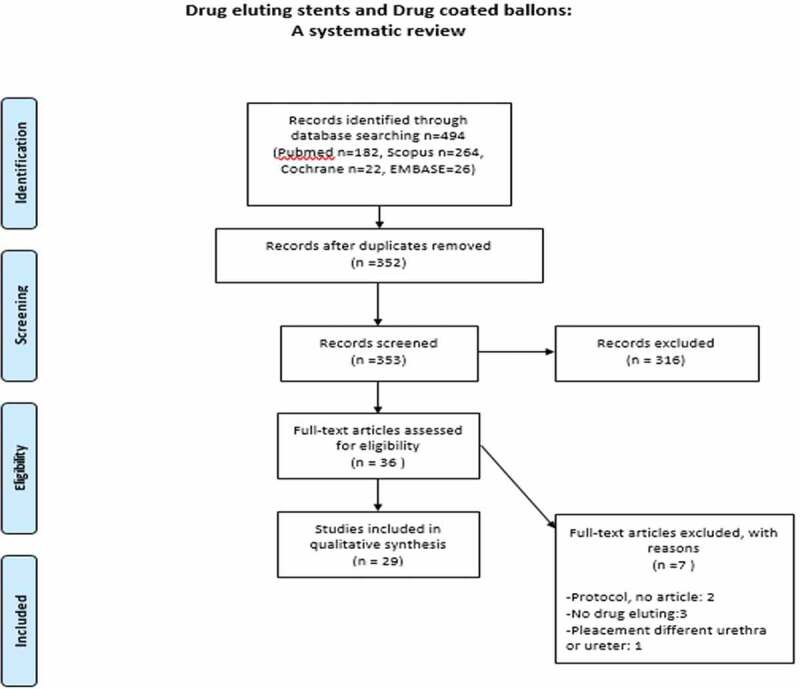
PRISMA flow chart of the systematic review

We conducted a systematic review according to the Preferred Reporting Items for Systematic Reviews and Meta-Analyses (PRISMA) statement. PubMed, Scopus and Cochrane Library online databases were searched in February 2019 for all relevant articles with no language restriction. The used search string was (elut* OR coat*) AND (stent OR balloon) AND (ureter* OR urethra*). The study was registered in International Prospective Register of Systematic Reviews (PROSPERO) with the reference number CRD42019121726 (Figure 1). The eligibility criteria are presented in Table 1.

**Table 1.** Eligibility criteria of the systematic review

Eligibility criteria
Human (male and female) or animal
Placement of DES or dilatation with DCB for investigating their potential use in the urinary tract for the management of ureteric or urethral pathologies.
Clinical trial or experimental studies
No restriction in date of publication
No language restriction
Articles in peer-reviewed journals and abstracts from major congresses (EAU, WCE, AUA, SIU) – same studies as above

EAU, European Association of Urology; SIU, Société Internationale d’Urologie; WCE, World Congress of Endourology.

### Data extraction

After de-duplication, the selected articles were screened by two authors (S.V., C.A.) independently according their titles and abstracts. Then, the full texts of the potentially eligible articles were retrieved and scrutinised using a standardised form. Disagreements were solved by consulting a supervisor (P.K.).

### Types of study design included

All studies, either experimental or clinical, which included the placement of DES/DCS or dilatation with a DCB for investigating their potential use in the urinary tract for the management of ureteric or urethral pathologies, were included.

### Population included

The population included in the clinical trials were humans (male and female), who underwent a stent insertion based on indications, and animals.

### Types of outcomes

The primary endpoint was to review the literature about the current and potential use of DES/DCS and DCB in urology.

## Results

### Search results

A total of 485 publications were screened for eligibility and 29 articles were eventually included in the systematic review (Figure 1).

### Study and population characteristics

Table 2 [9–17], Table 3 [3,18–36] and Table 4 [3,33] show a summary of the included studies. The available studies were published over a period of 18 years (2000–2019).

**Table 2.** Comparison of the experimental (*in vitro*) trials of DES/DCS

Reference	Aim/primary endpoint	Intervention	Results
Antimisiaris *et al*., 2000 [9]	*In vitro* preparation of liposome-covered metal stents and loading of liposomal drug formulations that will slowly release the drug in the vicinity of the stent.	Apply to pieces of stent a large multi-lamellar liposomes empty or entrapping the corticosteroid anti-inflamatory-drug	A mean (SD) 39.11 (6.8)% of the lipid and 50.84 (5.48)% of the drug was released from the stent pieces during 48 h incubation in the presence of artifical urine.
Cadieux *et al*., 2006 [12]	Test of the effects of triclosan-impregnated stent segments on the growth and survival of *Proteus mirabilis*	Instillation with 1 × 10^6^ *P. mirabilis* 296 Randomised groups, intravesical stent: -Triclosan -Optima® -Percuflex Plus® Urine culture: days 1, 3 and 7. Day 7: Incrustation and viable organisms in stents.	Urine culture: significantly less *P. mirabilis* in the triclosan group than in the Percuflex Plus group at all time points and in the Optima group on days 3 and 7
Barros et al., 2015 [10]	The evaluation of the *in vitro* elution profile of ketoprofen impregnated in the biodegradable ureteric stent during its degradation	To impregnate with ketoprofen the biodegradable ureteric stents with each formulation: alginate-based, gellan gum-based	Ketoprofen-impregnated stents were able to the release ketoprofen in the first 72 h in artificial urine solution.
Barros et al., 2017 [11]	The use of an *ex vivo* porcine model to assess the permeability of the anti-cancer drugs (PTX, doxorubicin) delivered from a BUS across porcine ureter	The permeability of the anti-cancer drugs (PTX, doxorubicin) alone or released from the developed BUS	PTX and doxorubicin drugs released from the BUS were able to remain in the *ex vivo* ureter and only a small amount of the drugs can cross the different permeable membranes with a permeability of 3% for PTX and 11% for doxorubicin. The estimated amount of PTX remains in the *ex vivo* ureter tissue and was shown to be effective against the cancer cells and did not affect the non-cancer cells.
Chew *et al*., 2006 [13]	The bactericidal and bacteriostatic effect of a triclosan-eluting ureteric stent against common bacterial uropathogens in an *in vitro* setting	Control stent and eluting stent with triclosan were suspended in artificial urine with bacterial pathogens to assess growth, virulence-promoter activity, and bacterial adherence	Triclosan stents had significantly fewer adherent viable bacteria than control stents. Growth was inhibited in a dose-dependent fashion by triclosan eluate in all strains except *P. aeruginosa* and *Ent. faecalis*
Elayarajah *et al*., 2011 [14]	Evaluation of ureteric stents made of silicone impregnated with one or more antimicrobial agents (ofloxacin and ornidazole) to inhibit the growth of different bacterial pathogens that colonise the device surface	Stent pieces were impregnated in a polymer mixed antibiotic solution (ofloxacin and ornidazole) for uniform surface coating (drug-carrier-coated stents) to inhibit the growth of different bacterial pathogens that colonise the device surface	In qualitative test, the zone of inhibition around the coated stents showed sensitivity against the clinical isolates. In quantitative test, the number of adhered bacteria on the surface of coated stents was reduced to a significant level (*P* < 0.05).
Johnson *et al*., 2010 [15]	Comparison of commercially available, antibiotic-coated Foley catheters regarding activity, comparative potency and effect durability	An inhibition zone assay (diffusible inhibition) and an adherence assay was used to assess the inhibitory effect of coated urethral catheters, 2 with silver and 1 with nitrofurazone	The nitrofurazone-coated catheter showed the greatest and most durable (through day 5) inhibitory activity. One of the 2 silver-coated catheters showed sparse but measurable inhibition zone activity on day 1, but not thereafter and no statistically significant activity on adherence assay. The other lacked detectable activity using either test system.
Kotsar *et al*., 2009 [16]	To assess the degradation process and the biocompatibility of biodegradable urethral DES.	The effect of cytokines and other inflammatory mediators on control stents (bacterial lipopolysaccharide as a positive control) and biodegradable stent material (poly-96 L/4D-lactic acid [PLA]) using the human cytokine antibody array	The increase in the production of inflamatory mediators with the PLA-stent material was smaller than in the cells treated with lipopolysaccharide
Ma *et al*., 2016 [17]	To assess the degradation process and the biocompatibility of biodegradable ciprofloxacin-eluting ureteric stents.	Poly(L-lactide-*co-ε*-caprolactone) degradation process and the biocompatibility of biodegradable ciprofloxacin-eluting ureteric stents. Ciprofloxacin-eluting ureteric stents were prepared by the dipping method.	Stage I: mainly controlled by chain scission instead of the weight loss or morphological changes of the coatings. Stage II: the release profile was dominated by erosion resulting from the hydrolysis reaction autocatalysed by acidic degradation residues. Ciprofloxacin-loaded coatings displayed a significant bacterial resistance against *E. coli* and *S. aureus* without obvious cytotoxicity to human foreskin fibroblasts.

BUS: biodegradable ureteric stent; PTX: paclitaxel.

**Table 3.** Comparison of the clinical (*in vivo*) trials of DES/DCS

Reference	Subjects	Site	Intervention	Method study	Results	Limitations
El-Nahas et al., 2018 [18]	126 patients	Ureter	RCT Postoperative stent placement Control group: 69 patients SSD-coated stent: 68 patients	At placement and at removal of the stent: Urine and stent culture, USSQ	No significant differences between groups in the incidence of bacteriuria and USSQ scores.	Short duration of stenting. Relatively small sample size
Cadieux et al., 2009 [19]	8 patients	Ureter	Each pacient: Control stent 3 months with antibiotics preoperative and postoperative After this: change to triclosan-eluting stent 3 months with no antibiotics	Stent removal and processing: cut into three equal-length sections (bladder, ureteric, and kidney) Encrustation and surface: blinded technician and air dried. stent-adherent organisms: brain heart Infusion agar susceptibility to triclosan was determined via duplicate plating on Mueller Hinton agar	Staphylococcus isolated more in control stents Enterococcus isolated more in triclosan stents. Fewer antibiotics were used during triclosan stenting, coinciding with a slightly higher number of positive urine cultures and significantly fewer symptomatic infections.	Not mentioned
Cauda *et.al*., 2008 [20]	5 patients with bilateral obstructions	Ureter	For each pacient heparin-coated JJ stent and a traditional polyurethane JJ stent for 1 month.	Before placement and after removal stents were analysed using FESEM, EDS, and Micro-IR. Comparison of thickness, extension, and composition of encrustation. Analysis of two heparin-coated stents at 10 and 12 months.	FESEM: significant differences between groups for encrustation thickness and extension. EDS and Micro-IR: in heparinised stents the encrustations were not as uniform and compact as those in the uncoated stents. 10–12 months: free of encrustations and had no changes in the heparin layer.	Small number of patients. Relatively arbitrary nature of how the encrustations were quantified.
Chew *et al*., 2010 [21]	92 Yorkshire pigs	Ureter	Randomisation in to 5 groups Control: non-DES + oral KL, *n* = 12 Autopsy at 2, 5, 15 days Control: non-DES, *n* = 20 15% KL-loaded stent, *n* = 20 13% KL-loaded stent, *n* = 20 7% KL-loaded stent, *n* = 20 Groups 2,3,4,5: Necropsies at 2, 5, 15, 30, 60 days	Percutaneous aspiration of urine and venipuncture were obtained immediately before the autopsy. KL levels were measured in plasma, urine, and tissue sampled from ureters, bladder, kidneys, and liver using HPLC. Remainder of the genitourinary organs and liver: fixed and processed for histopathological analysis and analysed for any abnormality by a veterinarian pathologist blinded to the treatment group	Majority of KL first 30 days. Highest levels of KL: Group 1. Highest levels of KL in ureter and bladder tissues: KL stent dose-dependent fashion. Gastric ulcerations: Group 1.	Not named
Cirioni *et al*., 2007 [22]	5 adult female Wistar rats	Ureter	2 × 10^7^ CFU/mL *S. aureus*: inoculated into the bladder Control group C0 without a bacterial challenge Challenged control group (C1) without antibiotic prophylaxis i. 10 mg/kg of body weight teicoplanin intraperitoneally immediately after stent implantation ii. RIP-coated (0.2-cm^2^) sterile stents iii. stents coated with intraperitoneal teicoplanin	Culturing serial 10-fold dilutions (0.1 mL) of the bacterial suspension on blood agar plates. 37°C for 48 h. Quantification: number of CFU/plate. Toxicity: presence of any drug-related adverse effects.	C0: None had microbiological evidence of stent infection. C1: all stents presented with infection with a mean (SD) of 6.6 (1.9) × 10^6^ CFU/mL. i. 3.8 (0.8) × 10^3^ CFU/mL ii. 6.7 (1.4) × 10^4^ CFU/mL (*P* < 0.05) iii. no bacterial counts (*P* < 0.001). None of the rats included in any group died or had any clinical evidence of drug-related adverse effects.	Not named
Han *et al*., 2018 [23]	6 dogs	Urethra	12 EW-7197-eluting NFCSs were placed in the proximal and distal urethras in each dog. Control stent (CS) group (*n* = 3) received NFCSs. Drug-stent (DS) group (*n* = 3) recived EW-7197 (1000 μg)-eluting NFCSs. All dogs were killed 8 weeks after stent placement	Urethrography: 4–8 weeks after stent’s placement. The histological samples were longitudinally sectioned at the three different portions of the segment with the stent. H&E and MT stains were used to study the samples. The items were submucosal inflammatory cell infiltration, the number of epithelial layers, the thickness of submucosal fibrosis, and the thickness of papillary projection.	Urethrographic analysis: mean luminal DS group > CS group at 4 and 8 weeks after stent placement (all *P* < 0.001) Histological examination: thicknesses of the papillary projection, thickness of submucosal fibrosis, number of epithelial layers, and degree of collagen DS group < CS group (all *P* < 0.001). Degree of inflammatory cell infiltration was not significantly different	Small sample size Quantitative analyses for the expression levels of TGF-t and its mediators were not possible. Some of the measurements in this study were obtained subjectively. Long-term effectiveness of the EW-7197-eluting NFCS was not evaluated.
Kallidonis *et al*., 2011 [24]	10 pigs and 6 rabbits	Ureter	A zotarolimus-eluting stents (ZES) and a bare metal stents (BMS) were inserted in each ureter and in the contralateral ureter as a control.	Evaluation Porcine: CT every week/4 weeks Rabbit: IVU every week/8 weeks. Renal scintigraphies were performed before stent insertion and during the third week in all animals. Optical coherence tomograph (OCT): evaluation of the luminal and intraluminal condition of the ureters with stents. Histological examination: glycol-methacrylate	Hyperplastic reaction in both groups. 7 porcine ureter: BMS completely obstructed. Porcine ureters with ZES stents without obstruction. 2 rabbit ureter: BMS completely obstructed. No rabbit ureter obstruction with ZES stent. OCT: hyperplastic reaction in the ureters with BMS > ureters with ZES.	The evaluation model was based on the comparison of the ZES with control BMS. The effect of these stents in cases of ureteric pathology was not evaluated. Lack of thorough statistical analysis
Kim *et al*., 2018 [37]	36 male Sprague-Dawley rats	Urethra	Randomisation equally: Group A: control biodegradable stents. Group B: stents coated with 90 μg/cm^2^ sirolimus Group C: stents coated with 450 μg/cm^2^ sirolimus Each group: 6 rats killed after 4 weeks, the remaining after 12 weeks.	Retrograde urethrography and histological examination (H&E stained slices)	Urethrographic and histological examination: Granulation tissue formation Groups B, C < A (*P* < 0.05 for all). No significant differences between B and C. Number of epithelial layers B > C at 4 weeks after stent placement (*P* < 0.001) Apoptosis C > B, A (*P* < 0.05)	Stents were placed in rats with normal urethras. Follow-up duration of 12 weeks may not have been sufficient to identify rebound effects. Physical stent changes not assessed
Kotsar *et al*., 2009 [16]	16 male rabbits	Urethra	4 groups: the stents inserted into the prostatic urethra. Drugs: indomethacin, dexamethasone and ciprofloxacine. 80 L/20D-PLGA stents without a drug coating served as controls. 4 rabbits in each group, were killed after 1 month	The urethra surrounding the stent was dissected from the rabbits *en bloc*. Tissue blocks: H&E following routine techniques Biodegradation process evaluated by optic microscopic analyses. Biological response eosoniphilia, acute inflammatory changes (polymorphonuclear leucocytes), chronic inflammatory changes (lymphocytes, plasma cells) and the amount of fibrosis	Control stents and the dexamethasone-eluting stents degraded totally during the follow-up period. Indomethacin- and ciprofloxacine-eluting stent groups, the degradation process was significantly delayed and they induced an increase in epithelial hyperplasia. All the stents induced eosinophilia. No significant differences in the intensity of acute or chronic inflammatory reactions and fibrosis.	Not named
Kotsar *et al*., 2012 [26]	24 male New Zealand White rabbits	Urethra	4 groups. Biodegradable braided pattern PLGA urethral stents coated with rasemic 50 L/50D PLA with two different concentrations of indomethacin were inserted into the prostatic urethra Half of the rabbits in each group were killed after 3 weeks and the other half after 3 months.	Histological analyses using H&E staining. Following biological response parameters: Inflammatory changes (neutrophil infiltration), chronic inflammatory changes (lymphocyte and plasma cell infiltration), foreign body reaction, fibrosis, calcification, and eosinophil infiltration The degradation process of the stent and the development of epithelial hyperplasia (polyposis) was evaluated by SEM.	SEM analysis revealed that indomethacin coating had no effect on the degradation process of the stents. Histological analyses at 3 weeks: indomethacin-eluting stents caused more calcification but no significant differences in other tissue reactions At 3 months: the indomethacin-eluting stents caused less inflammatory reaction and calcification compared with the control stents.	Not named
Kram et al., 2020 [28]	48 male 9-week-old Sprague–Dawley rats	Ureter	3 groups, all *n* = 16: 1. Dissection of the left ureter without uretero-ureteric anastomosis but blunt manipulation of the ureter 2. Transsection of the left ureter and end-to-end anastomosis with insertion of an uncoated ureteric stent 3. Transsection of the left ureter and end-to-end anastomosis with insertion of either a pacitaxel-coated ureteric stent.	Received daily intraperitoneal injections of 5-bromo-2-deoxyuridine the first 8 postoperative days, and were killed on day 28. Healing of the ureteric anastomosis and proliferation of urothelial cells was examined histologically (H&E staining) and immunohistochemically	Both types of stents showed inflammation, fibrosis and urothelial changes. Proliferation of urothelial cells was significantly lower in rats with PTX-coated stents compared to those with uncoated stents (labelling index 41.27 vs 51.58, *P* < 0.001).	The surgical procedure of end-to-end ureteric anastomosis is demanding. There are differences in morphology and in the degree of stent-induced reactions compared to humans
Krambeck et al., 2010 [29]	276 patients	Ureter	Randomisation was 1:1 to KL or control stent groups. Stents were removed by pull-string or cystoscopically. Patients were followed for 30 days after stent removal. Primary study endpoint was intervention for pain. Secondary endpoints included: intervention due to stent, pain medication use, VAS assessed pain, and patient sat isfaction assessed using a 5-point scale	Blood and urine samples were obtained for routine chemistry studies, serum KL levels and urine analysis on days 0, 1, 2, 4, 7 and 10 after placement, the day of stent removal, and days 2 and 30 after stent removal.	None of the safety cohort had detectable serum KL levels. No difference in primary (9.0% KL loaded vs 7.0% control, *P* > 0.66) or secondary (22.6% KL loaded vs 25.2% control, *P* > 0.67) intervention rates. Pain pill count at day 3: KL < Control (*P* < 0.05). use no or limited pain medications: KL > Control. Male KL > Female KL, Male and Female control (*P* < 0.05)	Subjective nature of ureteric stent pain and the lack of an adequate assessment tool.
Krane *et al*., 2011 [30]	12 Sprague-Dawley Rats	Urethra	Formation urethral scars via electrocautery. Groups (*n* = 6 in each): Group 1, coated with HF Group 2, uncoated First inserting the silicone catheter, then placing a 30-G needle transversely completely through the corpora spongiosum and catheter and applying 10 W of electrocautery to the needle for 3 s. At 2 weeks: killed and excision urethral tissue.	Histopathological analysis: MT stain or anti-alpha-1 collagen and then examined with bright-field microscopy. Drug levels: tissue specimens – >HF concentration analysis via spectrophotometry. Blood: HF concentration via spectrophotometry absorption at 243 nm on a standard curve.	Group 1: Local urethral concentration of HF was 10fold higher than serum concentration. Had no new type I collagen deposition after urethral injury. Gruop 2: had increased periurethral collagen type I deposition, typical of urethral stricture formation.	Small number of rats in each group. Systemic concentration was evaluated with spectroscopy and not HPLC, which may provide more accurate measurements.
Liatsikos et al., 2007 [31]	10 female pigs	Ureter	Randomly placed in either the right or left ureter in each of the 10 pigs 1. R-Stent (*n* = 10) 2. PTX-eluting coronary stent (*n* = 10) Percutaneous nephrostomy was performed under ultrasonographic guidance and the collecting system was visualised. Deployment of the stent was finalised: nephrostomy tube was capped.	Patency evaluation of uretericlumen: Radiograph of the nephrostomy tract, IVU, and virtual endoscopy at 24 h and 21 days after the initial procedure, respectively. Conventional ureteroscopy at 21 days. Pathology examination of ureter: same pathologist minimising possible bias.	21 day follow-up: Group 1: 5 completely occluded 2 partially stenosed Group 2: no occluded stent Pathology examination 21 days: Obstructed R-stents generated severe inflammation with metaplasia PTX-eluting MS generated a mild inflammatory response without hindering ureteric patency	The difference in length of the two stent groups
Lin *et al*., 2018 [32]	5 New Zealand White rabbits	Ureter	5 cm segment of the analgesic (KL and lidocaine)-eluting nanofibre-incorporated ureter stent was inserted. Urine and blood samples were collected 1, 3, 7, 14, 21, and 28 days.	Infrared spectra of the analgesic-loaded nanofibrous matrix were evaluated employing FTIR spectrometry. Elution behaviour characteristics of lidocaine and KL from the analgesic-eluting ureteric stents were evaluated using an *in vitro* release scheme	Analgesic-eluting ureteric stents could liberate high strengths of analgesics *in vitro* and *in vivo* for at least 50 and 30 days, respectively. The blood levels were much lower throughout the study period.	Results show a local and sustainable elution of KL and lidocaine from the nanofibrous matrix-loaded ureteric stents, the efficacy of released anal gesics is yet to be confirmed. The rabbit model is limited in terms of its ability to be reproduced in human studies.
Mendez-Probst *et al*., 2012 [34]	20 patients requiring short-term stenting (7–15 days)	Ureter	Group 1: *n* = 10, Percuflex Plus® non-eluting stent (control) Group 2: *n* = 10, Triumph® triclosan-eluting stent. Group 1: 3 days of levofloxacin prophylaxis (500 mg once daily) Group 2: did not recived antibiotic.	Midstream urine samples were collected from each subject just prior to both stent placement and removal. Urine culture Stents: cut into three equal length sections, brain heart infusion agar supplemented with 0.5% yeast extract. Stent isolates were sent for identification and standard antibiotic susceptibility testing	Stent placement after: Group 1: 9 ureteroscopic and 1extracorporeal shockwave lithotripsy Group 2: 8 ureteroscopic and 2 extracorporeal shockwave lithotrips No significant differences were observed for culture Group 2: reductions in lower flank pain scores during activity (58.1% reduction, *P* = 0.017) and urination (42.6%, *P* = 0.041), abdominal pain during activity (42.1%, *P* = 0.042) and urethral pain during urination (31.7%, *P* = 0.049).	Lack of an additional patient group containing subjects receiving both the triclosan eluting stent and postoperative antibiotics
Shin *et al*., 2005 [35]	20 male dogs	Urethra	20 PTX-eluting polyurethane-covered stents (drug stents) and 20 polyurethane-covered stents (control stents) were placed alternately between the proximal and distal urethra. Group 1: *n* = 10 killed after 4 weeks 1.1.: drug stent in proximal urethra. 1.2.: control stent in distal urethra. Group 2: *n* = 10 killed after 8 weeks 2.1.: control stent in proximal urethra. 2.2.: drug stent in distal urethra.	Group 1: retrograde urethrography after sacrifice to evaluate percentage diameter of stenosis Group 2: retrograde urethrography at 4 weeks and 8 weeks before sacrifice. The sectioned tissue samples were stained with H&E: number of epithelial layers, thickness of granulation tissue, thickness of the papillary projection, and degree of submucosal inflammatory cell infiltra tion	Strong tendency toward a lower percentage diameter of stenosis and numeric mean values of the four histological findings, which indicates less formation of tissue hyperplasia in the proximal urethra than in the distal urethra. Thickness of the papillary projection was significantly less in drug stents than in control stents in the proximal urethra in the 8-week group (*P* = 0.016)	The effective dose of PTX was not quantified to suppress the tissue response secondary to inserted stents. Retrograde urethrography results could be intrinsically inaccurate.
Wang et al., 2011 [36]	34 male New Zealand White rabbits with urethral strictures 4 male New Zealand White rabbits, without urethral strictures and stents	Urethra	Group 1: *n* = 17, control stents and strictures. Group 2: *n* = 17, drug PTX-stents and strictures Group 3: *n* = 4, without urethral strictures and stents	Changes in the stent and the urethral stent-area: examined with paediatric urethroscope at 4, 8, and 12 weeks after stent implantation. 12 weeks: Retrograde urethrography to assess the urethral lumen. Urodynamics: bladder capacity and urethral pressure. Histological analysis: H&E. Biological changes were assessed for DES and compared to control stent groups.	Retrograde urethrography and urodynamic results at 12 weeks showed no comparable differences among the three groups Urethroscopic and histological follow-up indicated that the drug stents had minimised the stent-related inflammatory responses, urothelial hyperplasia, and scar formation compared with the drug-free stents	Not named

CFU: colony-forming units; EDS: energy dispersive spectroscopy; FTIR: Fourier transform infrared; H&E: haematoxylin and eosin; HF: halofungione; HPLC: high-performance liquid chromatography; KL: ketorolac; Micro-IR: micro-infrared spectrophotometry; MT: Masson’s trichrome; NFCS: nanofibre-covered stent; PLA: poly-96 L/4D-lactic acid; PLGA: poly(lactic- co-glycolic acid; PTX: paclitaxel; RIP: RNAIII-inhibiting peptide; (FE)SEM: (field-emission) scanning electron microscopy; SSD: silver sulfadiazine; VAS: visual analogue scale.

**Table 4.** Comparison of the clinical trials (*in vivo*) of DCBs

Reference	Subjects	Site	Intervention	Method study	Results	Limitations
Barbalias et al., 2017 [3]	11 rabbits	Urethra	A. *n* = 2, balloon dilatation without PTX B. *n* = 3, PTX-coated balloon dilatation, urethra removed immediately C.*n* = 3, PTX-coated balloon dilatation, urethra removed after 24 h D.*n* = 3, PTX-coated balloon dilatation, urethra removed after 48 h	H&E and immunohistochemistry with polyclonal anti‐PTX antibody in posterior urethra	Existence of ruptures across the urethras of all the rabbits. A. No PTX, no inflammation B. PTX distributing in all layers, no inflammation C. PTX distributing in all layers, mild acute inflammation D. PTX distributing in all lauers, mild acute inflammation	The use of PTX-coated balloons is designed for the treatment of vascular diseases. The distribution of PTX was only evaluated in the posterior urethra, which is different from anterior urethra.
Liourdi *et al*., 2014 [33]	9 domestic pigs	Ureter	Right ureter of each pig: PTX-eluting balloon dilatation Left ureter of each pig: conventional balloon dilatation Ureter removal: Immediately After 12 h After 24 h	A, B, C: 2 samples from each ureter. 1 sample: investigated by nuclear magnetic resonance spectroscopy. 1 sample: histology and immunohistochemistry using a specific for PTX polyclonal antibody.	Groups B and C: reduced inflammation compared to controls. PTX present in urothelial, submucosal and muscle layer. Concentration of the PTX C < B Group A: PTX present in urothelium and submucosal layer.	Short period of investigation (24 h) Did not evaluate the distribution and effect of PTX in the case of ureteric stricture. The use of DCB is designed for vascular applications.

H&E: haematoxylin and eosin; PTX: paclitaxel.

There were eight *in vitro* trials summarised in Tables 2 and 21 clinical trials (*in vivo*) summarised in Table 4.

Two of the clinical trials involved DCB, of which one studied the use of DCB in the urethra [3], while the other investigated their use in the ureter [33]. In all, 19 clinical studies focussed on DES/DCS, of which 12 were about the use of DES/DCS in the ureter [12,18–22,24,28,29,31,32,34] and seven in the urethra [16,23,26,30,35–37].

Five clinical trials included humans [18–20,29,34]; three randomised controlled trials (RCTs) [18,29,34] and two comparative non-RCTs [19,20]. The remaining 16 clinical trials were performed using a variety of animal models (rabits, rats, pigs and dogs) [3,12,16,21–24,26,28,30–33,35–37].

### Comparisons of interventions results

A total of 10 studies tested anti-proliferative agents containing DES/DCS or DCB [3,11,24,28,30,31,33,35-37]. Of these, seven investigated paclitaxel [3,11,28,31,33,35,36], one zotarolimus [24], one sirolimus [37] and one halofugione (HF) [30].

Antibiotic agent-containing DES/DCS were tested in nine studies [12–15,17-19,22,34], of which four studied triclosan [12,13,19,34], two the use of quinolones [14,17], two separate articles studied the use of either teicoplanin [22] or nitrofurantoin [15], and one studied the use of silver sulfadiazine [18].

A total of eight studies investigated the release of anti-inflammatory agents by DES/DCS [10,16,21,23,26,27,29,32], of which four investigated ketorolac [10,21,29,32], three investigated indomethacin [16,26,27], and one a TGF-β type 1 receptor kinase inhibitor, EW-7197 [23]. Furthermore, another group studied heparin-eluting stents [20].

## Discussion

Given the success of DES/DCS and DCB in the prevention of re-stenosis in cardiology and the relevance to stricture formation and other relevant complications following endourological procedures [13], the present systematic review was performed to assess their investigation in our field. Overall, the included studies could be divided into three major categories according to the drug used: anti-cancer, antibiotic, and anti-inflammatory agents.

### DES/DCS containing-antibiotic agents

UTI is the most common hospital infection and is associated with the existence of foreign bodies in the urinary tract, e.g. catheters, stents, and nephrostomy tubes [15]. This is because the foreign body in the urinary tract forms a surface easily colonised by bacteria, which over time form a highly resistant biofilm [13–15,17]. In an attempt to address this, research has focussed on coating ureteric stents with antibiotics to prevent bacterial adhesion and reduce associated infection rates [13–15,17].

Triclosan was studied in four articles [12,13,19,34]. It is an antimicrobial agent that intervenes with fatty-acid synthesis and hence the integrity of the bacterial cell-wall [13]. Chew *et al*. [13] tested the efficacy of a triclosan-eluting stent in artificial urine against a variety of common uropathogens (*E. coli, E. faecalis, S. aureus, K. pneumoniae, P. mirabilis, P. aeruginosa*) assessing bacterial growth and adherence. They concluded that triclosan DES/DCS halted the growth of most of the uropathogens, except *P. aeruginosa*. Cadieux *et al*. [12] subsequently tested the efficacy of this stent using a rabbit UTI model in which the curls from triclosan DES/DCS and non-eluting controls were sutured in the bladder of rabbits, previously instilled with *P. mirabillis*. Culture of both urine and stent pieces revealed significantly reduced numbers in both samples from rabbits with a triclosan-eluting stent. In addition, the bladder of the triclosan group was less inflamed than the control group. Another study by Cadieux *et al*. [19] investigated the long-term effiacy of the triclosan-eluting stent in eight patients stented due to cancer, ureteric stricture or retroperitoneal fibrosis. After an initial 3-month control period with conventional stenting, the stents were replaced by triclosan DES/DCS for 3 months. Patients were monitored over both periods with urine cultures and were prescribed antibiotics as indicated by UTI symptoms. Overall, there was no significant difference in the number of adherent bacteria to either the trilosan-eluting or non-eluting stents or in urine cultures. However, the need for antibiotic therapy was lower with a triclosan stent in place suggesting lower symptomatic UTIs. In a separate RCT, Mendez *et al*. [34] studied the efficacy of the triclosan DES/DCS compared to a conventional stent in 20 patients (10 patients/group), who required short-term stenting. Overall, there was no difference in culture and encrustation between the groups; however, patients in the triclosan group reported lesser flank, abdominal or urethral pain during activity or urination.

Collectively, these studies indicate that despite promising effects with regard to the prevention of bacterial adhesion and development of UTI in *in vitro* and preclinical *in vivo* models, these same benefits were not seen in patients. That said, differences were observed in the use of antibiotics and lower overall dyscomfort, suggesting an effect on the development of symptomatic UTIs and patient discomfort. Despite these beneficial effects, this stent is no longer available on the market.

The antimicrobial effects of quinolones were studied in two articles [14,17]. Elayarajah *et al*. [14] used stents impregnated with a mixture of ofloxocin and ornidazole. Using a simplistic agar diffusion test they showed the impregnated stents to be effective at killing *E. coli* and *S. epidermidis*. In addition, they tested efficacy against preventing bacterial adhesion in artificial urine and found the number on the DES/DCS to be significantly lower compared to the conventional stent. Ma *et al*. [17] studied the characteristics and effiacy of three ciprofloxacin DES/DCS, differing in the composition of the antibiotic carrier. The stents remained in artificial urine for 120 days, over which time the coating degradation, antibiotic-release profile, the anti-bacterial activity, and cytotoxicity were assessed. It was found that ciprofloxacin release occured in three phases and was highly dependent on the degradation activity of the coating, except from the first 7 days (‘burst stage’). An inital burst release of antibiotics is important to ensure high enough concentrations to kill any introduced bacterial loads and prevent bacterial adhesion, colonisation and subsequent biofilm formation. Getting this amount right is critical, as the release of sub-optimal concentrations, especially over long periods of time, has a significant risk for inducing resistance. In general, it was shown that ciprofloxacin DES/DCS have good antibacterial activity against *S. aureus* and *E. coli*, and no cytotoxicity against human foreskin fibroblasts.

Johnson *et al*. [15], on the other hand, studied the antimicrobial effects of urethral catheters coated with silver and nitrofurazone by using an inhibition zone and adherence assay. They compared two silver-coated and one nitrofurazone-coated marketed urethral catheters according to the inhibitory effect against *E. coli* (extended spectrum cephalosporin resistant and susceptible strains) and a *P. aeruginosa*. The inhibition activity was greater and lasted longer with the nitrofurazone-coated catheter for all *E. coli* strains. The silver-coated catheter showed no statistically significant inhibition, while none of the three catheters had any effect against *P. aeruginosa*. Moreover, El-Nahas *et al*. [18] conducted an RCT comparing a silver sulfadiazine DCS to conventional stents in 126 patients after endoscopic lithotripsy. The authors compared the results from urine cultures collected before and after stenting (at removal), as well as the stenting-related symptoms using the Ureteric Stent Symptoms Questionaire (USSQ). The study concluded that there was no difference between the two groups regarding urine cultures and the USSQ scores. It is interesting that silver, a known antimicrobial currently used throughout medicine, was ineffective against both *E. coli* and *P. aeruginosa*, especially as it is known to be effective against these species. This may indicate that the concentration of silver released from these stents was not high enough to effectively kill the bacteria. This illustrates that while the ability to release a given agent from the surface of devices is important, it must reach high enough concentrations in the surrounding medium to be promising clincially.

Cirioni *et al*. [22] used a different appraoch, developing a stent that released RNAIII-inhibiting peptide, which inhibits the formation of staphylococcal biofilm and the production of toxins, with or without teicoplanin, and tested the efficacy in a rat model of UTI. Interestingly, they found the combination of RNAIII-inhibiting peptide and teicoplanin to decrease the colonisation drastically with no bacterial counts (*P <* 0.001) in urine culture.

An experiment to reduce encrustation was conducted by Cauda *et al*. [20], who did not use antibiotic agents, but heparin DES/DCS. Heparin is highly negatively charged and believed to prevent encrustation by repelling negatively charged crystals. They inserted a heparin DES/DCS and a conventional stent in five patients with bilateral obstruction and showed that the encrustation on heparinised stents were not as thick and extensive as the encrustation on the conventional one. Subsequent *in vitro* studies performed by Lange *et al*. [38] showed no significant difference in bacterial adhesion between the heparin-coated and -uncoated stents.

### Anti-inflammatory agent containing DES/DCS

One of the most common complications of indwelling stents is pain and discomfort felt by the patient. As a result, several studies have investigated the release of anti-inflammatory drugs by coating of stents with these agents [10,13,29].

Ketorolac, which is a NSAID, was tested in four studies [10,21,29,32]. Barros *et al*. [10] tested the release profile of ketolorac from two different types of biodegradable stents, in artificial urine. Ketorolac was found to be released within the first 72 h, which is the desirable duration in the case of postoperative stent insertion, where on average oedema resolves within 72 h.

Furthermore, the pharmakokinetics of ketorolac-eluting stents were studied in a porcine model by Chew *et al*. [21], by measuring the distribution of the drug in plasma, urine and relevant tissues in pigs receiving the drug orally or via the insertion of a ureteric stent containing either 15%, 13% or 7% of the drug. Overall, most of the drug was released within 30 days and the highest levels of the drug were found in the ureteric and bladder tissues in the stented groups in a dose-dependent fashion. Lin *et al*. [32] studied the delivery of lidocain and ketorolac of a DES/DCS *in vitro* and *in vivo* in rabbit models. They showed that the DES/DCS was able to obtain high doses of analgesic for at least 50 days *in vitro* and 30 days *in vivo*. Krambeck *et al*. [29] went one step further and performed a RCT on post-ureteroscopy patients. The RCT included 276 patients who underwent ureteroscopy due to stone disease or for diagnostic reasons. The primary endpoint of this RCT was pain, defined as unscheduled Emergency Room visits, alteration in pain management and early removal of the stent, while secondary endpoints included intervention due to the stents, visual analogue scale pain assessment, medication use, satisfaction of patient and plasma concentration of ketorolac in the group with DES/DCS. None of the plasma samples taken from patients with DES/DCS had detectable levels of the drug, while differences in pain were identified only in male participants aged <45 years. This overall negative result is likely attributed to the fact that despite promising results in preclinical *in vitro* and *in vivo* large animal studies, not enough drug reached relevant tissues to elicit an effect. The sustained release of a high enough drug concentration to achieve effective concentrations within relevant tissues to have an effect remains a challenge in the urinary tract and will need to be overcome before DES can become a reality in urology.

Aside from urethral DES, a few studies have investigated the use of drug elution to address strcture formation in the urethra. Indomethacin-eluting urethral stents were investigated by Kotsar *et al*. [16,26,27] in three studies. The first study investigated the degradation pattern of an absorbable urethral stent eluting indomethacin, dexamethasone, and ciprofloxacin. The stents were inserted in the posterior urethra of 16 rabbits, which were killed after 1 month at which point the urethras were harvested and histologically examined. The control and dexamethasone-coated stents were totally absorbed, while indomethacine and ciprofloxacine delayed the degradation process of the stent and caused epithelial hyperplasia. Histologically, there was no difference in either acute or chronic inflammation, or fibrosis [16]. In a subsequent study, they investigated the production of cytokines and other inflammatory mediators caused by absorbable urethral DRE *in vitro* and *in vivo*. The eluted compounds were indomethacin, dexamethasone, and simvastatin. The stents were inserted in the posterior urethra of 18 rabbits, which were killed at 3 weeks or 3 months. Overall, only dexamethasone-eluting stents produced a significantly greater reaction than the control group, while at 3 months the reaction was resolved in all groups. Based on these results, they concluded that the drug elution on absorbable stents does not intervene significantly with the degradation process of the stent and is considered safe [27]. In the last study, the group investigated the cytokine profiles induced by an absorbable indomethacin-eluting stent *in vitro* and *in vivo* in the rabbit urethra. Initial *in vitro* tests showed that indomethacin-eluting stents reduced the production of monocyte chemoattractant protein 1 (MCP-1) and RANTES (regulated on activation, normal T-cell expressed and secreted), while it had no influence on TGF-β production. Subsequent *in vivo* studies showed that at 3 months it caused less inflammation and calcification compared to the bare control stents, with no negative effects of drug elution on the degradation process of the stent [26]. These absorbable urethral stents might be useful after a urethrotomy to reduce the chance of re-stenosis, but further testing is mandatory.

Moreover, Han *et al*. [23] tested a nano-fibre covered self-expandable stent coated with EW-7197, which constitutes a TGF-β type 1 tyrosine kinase inhibitor, in a canine model over an 8-week period. The overall goal was to compare granulation tissue formation between DES/DCS and the control stent. Dogs with the DES/DCS had larger urethral luminal diameters and the pathology report revealed that in the DES/DCS group the papillary projection and submucosal fibrosis were less thick and the number of epithelial layers, as well as the degree of collagen deposition, was lower than in the control group. In addition, Krane *et al*. [30] studied the use of HF, a collagen type I inhibitor, eluting catheters in the urethra of rats following the creation of a stricture using electrocautery. Interestingly, no new type I collagen was formed in the group with HF-eluting catheters.

### DES/DCB and DCB containing anti-proliferative agent

DES/DCS releasing an anti-proliferative agent have been used with great success in interventional cardiology due to their ability to stop cell proliferation and hence the formation of hyperplastic or fibrotic tissue [31,35]. Paclitaxel is the most tested drug in this category [3,11,28,31,33,35,36]. Barros *et al*. [11] did an experimental trial with biodegradable ureteric stents eluting paclitaxel and doxorubicine to investigate the permeability of the drugs in three different membrane models: polyethersulfone membrane, human umbilical vein endothelial cells, and *ex vivo* porcine ureter. Overall, they found that the two drugs remained in the ureter and only a small proportion crossed all layers of the ureter. Kram *et al*. [28] studied the hyperplastic proliferation of the ureter and whether it can be prevented by the use of paclitaxel DES/DCS in a rat model in which a ureteroureterostomy was performed and either a conventional or a paclitaxel DES was inserted. The histological report stated that the proliferation on the anastomosis side was lower in the paclitaxel group (laballing index 41.27 vs 51.58, *P* < 0.001). Likewise, Liatsikos *et al*. [31] compared a conventional stent to a paclitaxel DES/DCS in the porcine ureter and concluded that after 21 days ureters with an indwelling paclitaxel DES/DCS remained more patent on urography due to the production of less inflammation or hyperplasia. Similar results were obtained by Shin *et al*. [35] in the canine urethra.

Wang *et al*. [36] tested a biodegradable paclitaxel DES/DCS in the urethra of rabbits, and observed that at 12 weeks indwelling time the stents were completely absorbed. In the DES/DCS group the urethral mucosa resembled normal mucosa, whereas in the control group, the urethra was tough and rigid.

In addition to DES/DCS, DCB have also been studied as a way to dilate urethral and ureteric strictures. Along these lines, Barbalias *et al*. [3] studied the distribution of paclitaxel in the urethral layers of rabbits. For this, the posterior urethra was dilatated and a paclitaxel-coated balloon inflated at the same location. Paclitaxel was found to be distributed througout the urethral tissue upon histological analysis. Similarly, Liourdi *et al*. [33] used the same setup in the ureter and found pactlitxel distributed in all layers of the ureter. While paclitaxel DES/DCS and DCB represent an interesting approach that has been validated *in vitro* and *in vivo*, it must be pointed out that this has been done in models that did not include a stricture. As a result further studies in more realistic *in vivo* or human clinical trials are required to determine usefulness of these devices in clinical practice.

Besides paclitataxel, other chemotherapeutic drugs have been used as well. Kallidonis *et al*. [24] studied a zotarolimus DES/DCS in pigs and rabbits, and showed that anti-cancer DES/DCS prevent obstruction by reducing inflammation and hyperplasia.

## Conclusions

There is no doubt that there is great potential for drug-coated/eluting devices to become useful at preventing complications associated with conventional stents, including pain and encrustation. Pre-clinical *in vitro* and *in vivo* studies have shown their ability to reduce inflammation, prevent re-stenosis, and improve pain as indicated by decreased use of anti-inflammatory drugs. However, three RCTs that included humans have shown ambiguous results. Therefore, human trials and testing in realistic clinical scenarios are still lacking and required to give insight into whether or not DES/DCS and DCB that have made a significant difference in cardiology will also do so in urology. One of the biggest challenges that will need to be achieved for DES/DCS and DCB to be effective is the sustained release of the drugs at concentrations high enough to overcome dilution in urine and elimination via urine flow and reach effective levels in the ureteric tissues.

## References

[1] Al-Aown A, Kyriazis I, Kallidonis P. Ureteral stents: new ideas, new designs. Therapeutic Advances in Urology. 2010;2(2):85–92. 21789086 10.1177/1756287210370699 PMC3126070

[2] Desai M, Vyas J, Ganpule A,et al.Balloon dilatation for male urethral strictures. “Revisited”. Urology Annals. 2013;5(4):245–248. 24311903 10.4103/0974-7796.120296 PMC3835981

[3] Barbalias D, Lappas G, Ravazoula P, et al. Evaluation of the distribution of paclitaxel after application of a paclitaxel-coated balloon in the rabbit urethra. Journal of Endourology. 2018;32(5):381–386. 29382215 10.1089/end.2017.0935

[4] Fattori R, Piva T. Drug-eluting stents in vascular intervention. The Lancet. 2003;361(9353):247–249. 10.1016/S0140-6736(03)12275-1 12547552

[5] Ni L, Chen H, Luo Z, et al. Bioresorbable vascular stents and drug-eluting stents in treatment of coronary heart disease: a meta-analysis. Journal of Cardiothoracic Surgery. 2020;15(1):26. 31992360 10.1186/s13019-020-1041-5 PMC6986072

[6] Stefanini GG, Holmes DR Jr. Drug-eluting coronary-artery stents. New England Journal of Medicine. 2013;368(3):254–265. 10.1056/NEJMra1210816 23323902

[7] Nestelberger T, Kaiser C, Jeger R. Drug-coated balloons in cardiovascular disease: benefits, challenges, and clinical applications. Expert Opinion on Drug Delivery. 2020;17(2):201–211. 31918593 10.1080/17425247.2020.1714590

[8] Kallidonis PS, Georgiopoulos IS, Kyriazis ID, et al. Drug-eluting metallic stents in urology. Indian J Urol. 2014;30(1):8–12. 24497674 10.4103/0970-1591.124198 PMC3897059

[9] Antimisiaris SG, Siablis D, Liatsikos E, et al. Liposome-Coated Metal Stents: an in Vitro Evaluation of Controlled-Release Modality in the Ureter. J Endourol. 2000;14(9):743–747. 11110569 10.1089/end.2000.14.743

[10] Barros AA, Oliveira C, Reis RL, et al. Ketoprofen-eluting biodegradable ureteral stents by CO_2_ impregnation: *in vitro* study. Int J Pharm. 2015;495(2):651–659. 26392243 10.1016/j.ijpharm.2015.08.040

[11] Barros AA, Oliveira C, Reis RL, et al. In Vitro and Ex Vivo Permeability Studies of Paclitaxel and Doxorubicin From Drug-Eluting Biodegradable Ureteral Stents. J Pharm Sci. 2017;106(6):1466–1474. 28257819 10.1016/j.xphs.2017.02.023

[12] Cadieux PA, Chew BH, Knudsen BE, et al. Triclosan loaded ureteral stents decrease proteus mirabilis 296 infection in a rabbit urinary tract infection model. J Urol. 2006;175(6):2331–2335. 16697868 10.1016/S0022-5347(06)00252-7

[13] Chew BH, Cadieux PA, Reid G, et al. Second Prize : in-Vitro Activity of Triclosan-Eluting Ureteral Stents against Common Bacterial Uropathogens. J Endourol. 2006;20(11):949–58. 17144870 10.1089/end.2006.20.949

[14] Elayarajah E, Rajendran R, Venkatrajah B, et al. Biopolymer tocopherol acetate as a drug carrier to prevent bacterial biofilm formation on silicone ureteral stents. Int J Pharm Sci Rev Res. 2011;7:96–103.

[15] Johnson JR, Johnston BD, Kuskowski MA, et al. In vitro activity of available antimicrobial coated foley catheters against escherichia coli, including strains resistant to extended spectrum cephalosporins. J Urol. 2010;184(6):2572–2577. 21030047 10.1016/j.juro.2010.07.032

[16] Kotsar A, Isotalo T, Uurto I, et al. Urethral in situ biocompatibility of new drug-eluting biodegradable stents: an experimental study in the rabbit. BJU International. 2009;103(8):1132–1135. 19040531 10.1111/j.1464-410X.2008.08203.x

[17] Ma X, Xiao Y, Xu H, et al. Preparation, degradation and in vitro release of ciprofloxacin-eluting ureteral stents for potential antibacterial application. Mater Sci Eng C Mater Biol Appl. 2016;66:92–99. 27207042 10.1016/j.msec.2016.04.072

[18] El-Nahas AR, Lachine M, Elsawy E, et al. A randomized controlled trial comparing antimicrobial (silver sulfadiazine)-coated ureteral stents with non-coated stents. Scand J Urol. 2018;52(1):76–80. 28931344 10.1080/21681805.2017.1376353

[19] Cadieux PA, Chew BH, Nott L, et al. Use of triclosan-eluting ureteral stents in patients with long-term stents. J Endourol. 2009;23(7):1187–1194. 19538062 10.1089/end.2008.0437

[20] Cauda F, Cauda V, Fiori C, et al. Heparin Coating on Ureteral Double J Stents Prevents Encrustations: an in Vivo Case Study. J Endourol. 2008;22(3):465–472. 18307380 10.1089/end.2007.0218

[21] Chew BH, Davoudi H, Li J, et al. An In Vivo Porcine Evaluation of the Safety, Bioavailability, and Tissue Penetration of a Ketorolac Drug-Eluting Ureteral Stent Designed to Improve Comfort. J Endourol. 2010;24(6):1023–1029. 20367085 10.1089/end.2009.0523

[22] Cirioni O, Ghiselli R, Minardi D, et al. RNAIII-inhibiting peptide affects biofilm formation in a rat model of staphylococcal ureteral stent infection. Antimicrob Agents Chemother. 2007;51(12):4518–4520. 17875996 10.1128/AAC.00808-07 PMC2167994

[23] Han K, Park J-H, Yang S-G, et al. EW-7197 eluting nano-fiber covered self-expandable metallic stent to prevent granulation tissue formation in a canine urethral model. PLoS One. 2018;13(2):e0192430. 29447198 10.1371/journal.pone.0192430 PMC5813937

[24] Kallidonis P, Kitrou P, Karnabatidis D, et al. Evaluation of zotarolimus-eluting metal stent in animal ureters. J Endourol. 2011;25(10):1661–16617. 21905851 10.1089/end.2011.0308

[25] Kim SW, Park NC, Lee SW, et al. Efficacy and safety of a fixed-dose combination therapy of tamsulosin and tadalafil for patients with lower urinary tract symptoms and erectile dysfunction: results of a randomized, double-blinded, active-controlled trial. J Sex Med. 2017;14(8):1018–1027. 28760246 10.1016/j.jsxm.2017.06.006

[26] Kotsar A, Nieminen R, Isotalo T, et al. Preclinical evaluation of new indomethacin-eluting biodegradable urethral stent. J Endourol. 2012;26(4):387–392. 22050507 10.1089/end.2011.0327

[27] Kotsar A, Nieminen R, Isotalo T, et al. Biocompatibility of new drug-eluting biodegradable urethral stent materials. Urology. 2010;75(1):229–234. 19647295 10.1016/j.urology.2009.03.016

[28] Kram W, Rebl H, Wyrwa R, et al. Paclitaxel-coated stents to prevent hyperplastic proliferation of ureteral tissue: from in vitro to in vivo. Urolithiasis. 2020;48(1):47–56. 30259058 10.1007/s00240-018-1081-7

[29] Krambeck AE, Walsh RS, Denstedt JD, et al. A novel drug eluting ureteral stent: a prospective, randomized, multicenter clinical trial to evaluate the safety and effectiveness of a ketorolac loaded ureteral stent. J Urol. 2010;183(3):1037–1042. 20092835 10.1016/j.juro.2009.11.035

[30] Krane LS, Gorbachinsky I, Sirintrapun J, et al. Halofuginone-coated urethral catheters prevent periurethral spongiofibrosis in a rat model of urethral injury. J Endourol. 2011;25(1):107–112. 21204688 10.1089/end.2010.0514

[31] Liatsikos EN, Karnabatidis D, Kagadis GC, et al. Application of paclitaxel-eluting metal mesh stents within the pig ureter: an experimental study. Eur Urol. 2007;51(1):217–223. 16814926 10.1016/j.eururo.2006.05.054

[32] Lin Y-C, Liu K-S, Lee D, et al. In Vivo and In Vitro Elution of Analgesics from Multilayered Poly(D,L)-lactide- co -glycolide Nanofibers Incorporated Ureteral Stents. J Nanomater. 2018;2018:4943210.

[33] Liourdi D, Kallidonis P, Kyriazis I, et al. Evaluation of the distribution of paclitaxel by immunohistochemistry and nuclear magnetic resonance spectroscopy after the application of a drug-eluting balloon in the porcine ureter. J Endourol. 2015;29(5):580–589. 25441059 10.1089/end.2014.0683

[34] Mendez-Probst CE, Goneau LW, MacDonald KW, et al. The use of triclosan eluting stents effectively reduces ureteral stent symptoms: a prospective randomized trial. BJU International. 2012;110(5):749–754. 22313688 10.1111/j.1464-410X.2011.10903.x

[35] Shin JH, Song H-Y, Choi CG, et al. Tissue Hyperplasia: influence of a Paclitaxel-eluting Covered Stent—Preliminary Study in a Canine Urethral Model. Radiology. 2005;234(2):438–444. 15671001 10.1148/radiol.2342040006

[36] Wang Z-X, Hong B-F, Zhang X, et al. New biodegradable drug-eluting stents for urethral strictures in a rabbit model. J Bioact Compat Pol. 2011;26(1):89–98.

[37] Kim KY, Park J-H, Kim DH, et al. Sirolimus-eluting Biodegradable Poly- l -Lactic Acid Stent to Suppress Granulation Tissue Formation in the Rat Urethra. Radiology. 2018;286(1):140–148. 28787263 10.1148/radiol.2017170414

[38] Lange D, Elwood CN, Choi K, et al. Uropathogen interaction with the surface of urological stents using different surface properties. J Urol. 2009;182(3):1194–1200. 19625060 10.1016/j.juro.2009.05.008

